# MASLD, diabetes and PMOS across the female life stages

**DOI:** 10.1007/s00125-026-06761-y

**Published:** 2026-06-05

**Authors:** Elisabet Stener-Victorin, Sofia Carlsson, Hannes Hagström, Nayere Taebnia

**Affiliations:** 1 Department of Physiology and Pharmacology, Karolinska Institutet, Stockholm, Sweden; 2 Institute of Environmental Medicine, Karolinska Institutet, Stockholm, Sweden; 3 Department of Medicine, Karolinska Institutet, Stockholm, Sweden; 4 Division of Hepatology, Department of Upper GI Diseases, Karolinska University Hospital, Stockholm, Sweden

**Keywords:** Adipose tissue, Liver, MASLD, Metabolic dysfunction-associated steatotic liver disease, PCOS, PMOS, Polycystic ovary syndrome, Polyendocrine metabolic ovarian syndrome, Review, Type 2 diabetes

## Abstract

Metabolic dysfunction-associated steatotic liver disease (MASLD) and type 2 diabetes, which affect approximately 38% and 10.5%, respectively, of adults globally, intersect critically in women through polyendocrine metabolic ovarian syndrome (PMOS), previously known as polycystic ovary syndrome (PCOS), a prevalent but under-recognised cardiometabolic disorder affecting 10–13% of reproductive-age women. Beyond its reproductive consequences, PMOS promotes hepatic disease through hyperandrogenism, insulin resistance and chronic inflammation. Androgens directly upregulate hepatic lipogenic gene expression, activate hepatic stellate cells via androgen receptor signalling, and suppress sex hormone-binding globulin, creating a pro-fibrotic, lipotoxic hepatic environment. MASLD shares pathophysiological mechanisms with type 2 diabetes and PMOS, including adipose tissue dysfunction, adipokine dysregulation, hepatic lipid dysregulation and mitochondrial impairment. These shared mechanisms are further modulated by hormonal transitions during adolescence, pregnancy and menopause, with each representing a critical window of vulnerability. This narrative review consolidates current epidemiological and mechanistic insights linking MASLD, type 2 diabetes and PMOS in women, highlights the relevance of experimental models for elucidating shared disease pathways, and informs life stage-tailored screening and early intervention strategies to reduce long-term cardiometabolic and liver-related morbidity in affected women.

**Graphical Abstract:**

**Figure:**
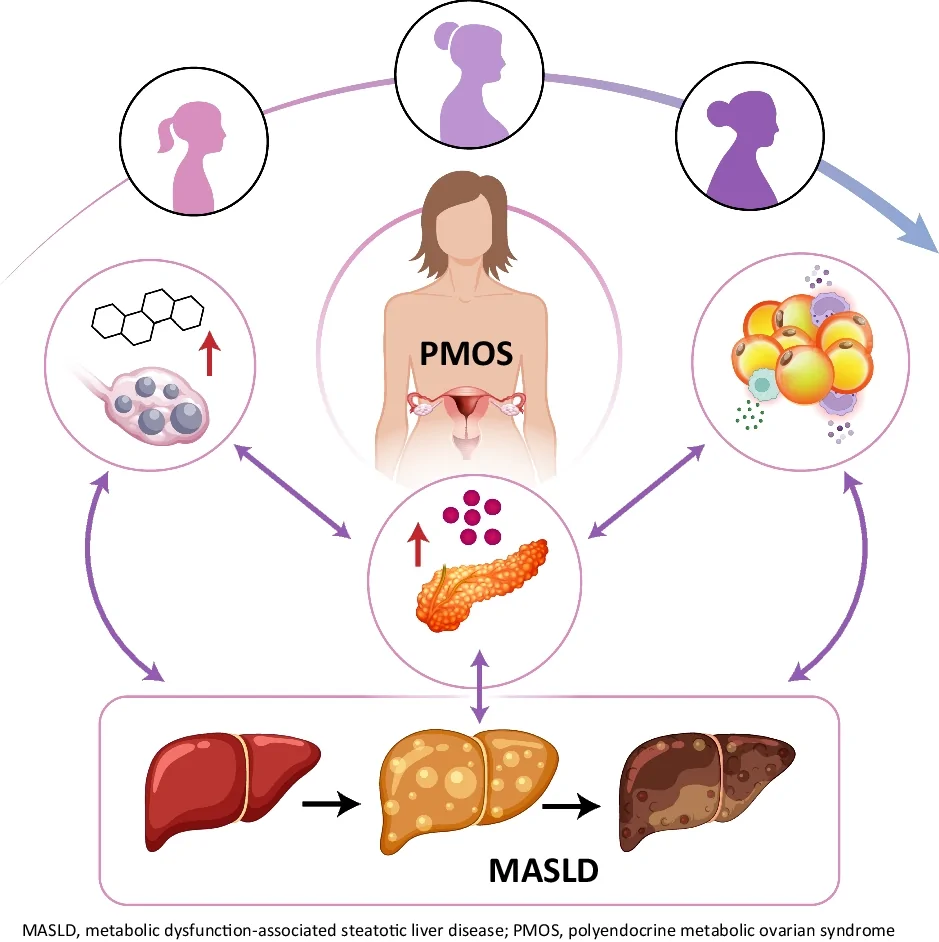

**Supplementary Information:**

The online version contains a slideset of the figures for download available at https://doi.org/10.1007/s00125-026-06761-y.

## Introduction

Metabolic dysfunction-associated steatotic liver disease (MASLD), which affects 30–40% of the global population [1], is increasingly prevalent in women and represents a major chronic disease burden throughout the female lifespan [2, 3]. MASLD is characterised by excess hepatic fat accumulation (steatosis) and can progress over decades to advanced liver disease through the accumulation of fibrosis, ultimately resulting in cirrhosis and an increased risk of hepatocellular carcinoma [4]. Beyond liver-related outcomes, MASLD is associated with type 1 diabetes [5], type 2 diabetes [4], CVD [6], chronic kidney disease and extrahepatic cancers [7].

MASLD is generally more prevalent in men [3]; however, this sex difference disappears in individuals with type 2 diabetes, suggesting loss of the typical female protection against hepatic steatosis [8, 9]. Several female-specific conditions further modify MASLD risk throughout life. Polyendocrine metabolic ovarian syndrome (PMOS) [10], previously known as polycystic ovary syndrome (PCOS), is characterised by hyperandrogenism, hyperinsulinaemia, insulin resistance and dyslipidaemia and significantly increases the risk of early-onset MASLD, type 2 diabetes and CVD [11–16]. Renaming PCOS to PMOS acknowledges the substantial cardiometabolic burden of the condition and represents one of the most comprehensive disease renaming processes documented to date [10].

Emerging evidence suggests a link between early menarche (before age 12 years) and MASLD later in life [17]. Pregnancy represents another critical window, as MASLD is associated with a higher risk of gestational diabetes mellitus (GDM), which in turn substantially increases the risk of future type 2 diabetes [18]. Menopause also represents a key transition, during which declining oestrogen levels are accompanied by increased central adiposity and ectopic fat accumulation, thereby increasing the risk of MASLD and type 2 diabetes [19].

Notably, the prevalences of MASLD, type 2 diabetes, PMOS and GDM have risen in parallel over recent decades, largely reflecting the global increase in overweight and obesity [20–23], a central shared risk factor underlying all of these conditions. Together, these converging trends represent a growing threat to women’s health, with important implications for long-term morbidity and mortality.

Recent reviews have examined MASLD in relation to endocrine disorders, as well as sex differences in MASLD, often focusing on specific domains such as pathophysiology or metabolic risk in selected populations [3, 24, 25]. While informative, these studies typically address individual components in isolation. In contrast, this review integrates MASLD, type 2 diabetes and type 1 diabetes (where relevant to MASLD risk), highlighting how female-specific conditions and life stages, particularly PMOS, early menarche, pregnancy and menopause, contribute to MASLD risk. It also positions PMOS, type 2 diabetes and MASLD within a shared mechanistic continuum across key female life stages and incorporates mechanistic pathways and emerging experimental models to provide a more comprehensive, translational perspective on female-specific MASLD risk.

## Epidemiology of MASLD, PMOS and diabetes

There is a bidirectional link between MASLD and type 2 diabetes. Meta-analyses show a two- to threefold higher risk of type 2 diabetes among individuals with MASLD [26], and global estimates suggest that approximately 65% of people with type 2 diabetes have MASLD, with the highest prevalences reported in Eastern Europe and the Middle East and the lowest in Africa [8]. Type 2 diabetes may, in turn, accelerate MASLD progression, and people with type 2 diabetes have a roughly twofold increased risk of cirrhosis compared with the general population [8]. One study has also suggested a higher rate of some extrahepatic cancers in women with MASLD than in men with MASLD, compared with matched control participants [27]. Selected key epidemiological studies supporting these associations are summarised in Table 1.

**Table 1:** Selected epidemiological studies linking MASLD, PMOS and diabetes in women

Topic	Study (year)	Design	Setting	Main finding
MASLD → type 2 diabetes	Mantovani (2021) [26]	Meta-analysis	International (multiple cohorts)	MASLD is associated with a two- to threefold higher risk of incident type 2 diabetes
MASLD in type 2 diabetes	Younossi (2024) [8]	Meta-analysis/review	Global	Approximately 65% of individuals with type 2 diabetes have MASLD
PMOS → type 2 diabetes	Glintborg (2024) [14]	Nationwide Nordic cohort	Denmark, Finland, Sweden	There is an approximately threefold higher risk of type 2 diabetes in women with PMOS
PMOS → MASLD	Shengir (2021) [30]	Meta-analysis	International (multiple cohorts)	There is an approximately twofold higher risk of MASLD in women with PMOS
MASLD → GDM	Sarkar (2021) [32]	Population-based study	USA (national inpatient data)	MASLD is associated with an approximately threefold increased risk of GDM
GDM + MASLD → type 2 diabetes	Cho (2023) [18]	Cohort study	South Korea (health screening cohort)	MASLD and GDM interact to increase the risk of type 2 diabetes by approximately sixfold
MASLD in type 1 diabetes	de Vries (2020) [33]	Meta-analysis	International (multiple cohorts)	MASLD prevalence is approximately 22% in type 1 diabetes

Effect estimates are presented as approximate RRs or HRs as reported in the original studies or meta-analyses

The reported prevalence of PMOS varies widely across populations, ranging from 4% to over 25%, likely reflecting both true population differences and heterogeneity in diagnostic criteria, subtypes, study design and sample sizes [21, 22, 28]. Women with PMOS have an approximately twofold higher risk of MASLD [3, 29], with the greatest risk observed in South American and Middle Eastern populations, an intermediate risk seen in Asian populations and the lowest risk found in European populations [30]. PMOS is also associated with a threefold increased risk of type 2 diabetes [14]. Emerging evidence suggests that PMOS may confer a particularly high MASLD risk among young women living with overweight or obesity and type 2 diabetes [31], and that PMOS and type 2 diabetes together may accelerate progression from steatosis to steatohepatitis and advanced fibrosis [8, 32].

**Fig. 1:**
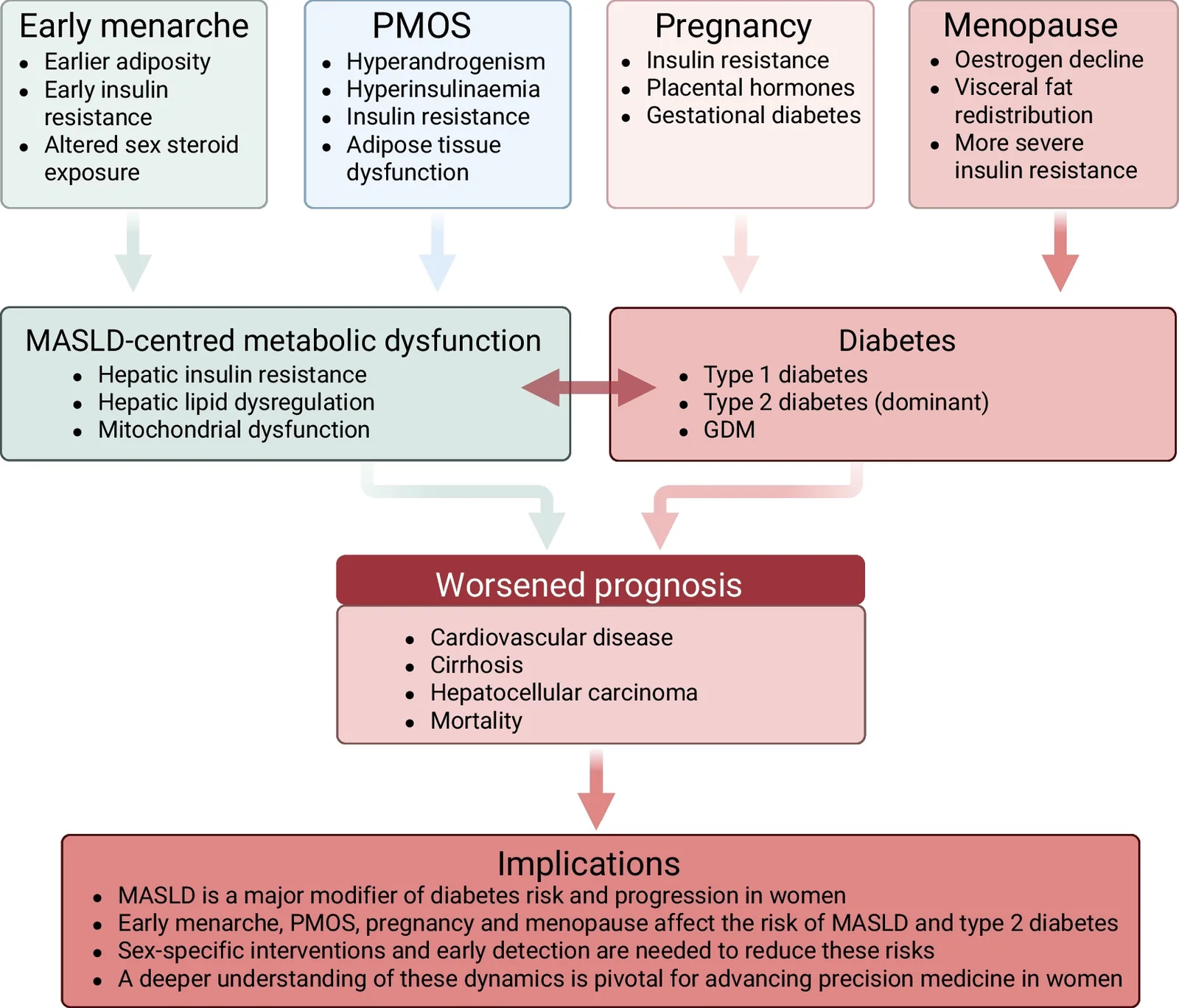
Conceptual framework linking female reproductive stages with insulin resistance, MASLD, diabetes and cardiometabolic risk. Key reproductive stages and conditions—early menarche, PMOS, pregnancy and menopause—drive hormonal and metabolic changes that influence insulin resistance and the development of MASLD. MASLD acts as a central metabolic hub with bidirectional relationships with diabetes, with both conditions jointly promoting adverse cardiometabolic outcomes later in life. The framework underscores the need to incorporate female-specific biology into risk stratification and prevention strategies for MASLD and diabetes. This figure is available as part of a downloadable slideset

MASLD is also increasingly recognised among individuals with type 1 diabetes. A meta-analysis estimated a MASLD prevalence of approximately 22% in adults with type 1 diabetes [33]. Although type 1 and type 2 diabetes differ fundamentally in their pathophysiology, with type 1 diabetes characterised by autoimmune beta cell destruction, their metabolic profiles increasingly overlap, as a growing proportion of individuals with type 1 diabetes are living with overweight and are insulin resistant. MASLD is associated with higher risks of microvascular and macrovascular complications in both type 1 and type 2 diabetes [34] and with increased overall mortality in type 2 diabetes [35] (Fig. 1).

## Genetic basis of MASLD, PMOS and type 2 diabetes

The genetic basis of MASLD, PMOS and type 2 diabetes in women involves both shared and distinct loci that converge on insulin resistance, lipid metabolism and reproductive and endocrine regulation and which may help explain inter-individual variability and sex-specific trajectories. MASLD is most strongly associated with the *PNPLA3* rs738409[G] variant encoding the I148M substitution, which disrupts hepatic lipid homeostasis through impaired lipolysis, altered triacylglycerol remodelling and mitochondrial redox shifts [36, 37]. The deleterious effects of this variant are amplified in women through oestrogen receptor (ER)-mediated hepatic PNPLA3 expression, especially after menopause, when the loss of oestrogen’s protective influence increases disease risk via upregulation of PNPLA3 [38, 39]. *TM6SF2* rs58542926 impairs hepatic VLDL secretion, promoting steatosis while reducing circulating LDL-cholesterol [40], and *GCKR* rs1260326 enhances hepatic glycolysis and lipogenesis, linking fat accumulation to improved insulin sensitivity [41]. *MBOAT7* rs641738 promotes steatosis via phosphatidylinositol turnover [42], while *GPAM* rs2792751 increases triacylglycerol synthesis and cirrhosis risk [43]. Conversely, protective variants in *HSD17B13* (rs72613567) and *MTARC1* (rs2642438, A165T) reduce the risk of progression to advanced liver disease [44, 45]. These loci illustrate how pathways in hepatic lipid handling intersect with systemic insulin resistance, an overlap that becomes particularly relevant when considering PMOS and type 2 diabetes risk in women.

PMOS is highly heritable, with twin studies estimating heritability of up to 70% [46, 47]. A recent multi-ancestry genome-wide association study (GWAS) identified 94 loci, including *AMH*, *FSHR*, *LHCGR*, *DENND1A* and *INSR*, linking ovarian folliculogenesis to metabolic dysfunction [48]. Genetically predicted childhood body size independently contributes to PMOS risk [49], correlating with higher childhood BMI and adiposity [50]. Mendelian randomisation supports a causal contribution of increased BMI (and in some analyses type 2 diabetes liability) to PMOS risk, while evidence for reverse causality is inconsistent [48, 51, 52]. Triacylglycerol associations appear to be ancestry specific and are significant only in European women with PMOS [48]. Notably, higher MASLD genetic risk scores are positively associated with PMOS prevalence (OR 1.10; 95% CI 1.02, 1.18), suggesting shared metabolic vulnerability rather than a strictly unidirectional relationship [53]. Moreover, Mendelian randomisation evidence indicates that genetically predicted testosterone elevation causally increases type 2 diabetes risk in women (OR 1.51) and is associated with PMOS-related traits, while exerting protective cardiometabolic effects in men, underscoring the fundamentally sex-specific consequences of androgen signalling [54]. Elevated testosterone also increases the risks of ER-positive breast and endometrial cancer, reflecting tissue-specific androgen–oestrogen interactions [54]. Shared variants in *GCKR*, *FTO*, *TCF7L2*, *PNPLA3* and *TM6SF2* link PMOS, type 2 diabetes and MASLD, highlighting overlapping aetiology [55, 56].

The convergence of these diseases in women is shaped not only by genetic overlap but also by sex-specific modifiers. Oestrogen and testosterone play central roles in modulating gene expression in hepatic and ovarian tissues, affecting disease penetrance and progression. Genetic pleiotropy, such as in *GCKR* and *PPARG*, supports the concept of a shared cardiometabolic–reproductive axis. Emerging methods such as partitioned polygenic risk scores enable phenotype-specific stratification and could help provide a precision medicine framework across these interconnected disorders.

## Developmental and epigenetic mechanisms linking MASLD, PMOS and diabetes pathogenesis

Environmental and epigenetic mechanisms critically contribute to the pathogenesis of MASLD [57], PMOS [58] and type 2 diabetes [59, 60]. In MASLD, maternal under- and overnutrition, endocrine-disrupting chemicals and air pollutants induce hepatic steatosis via epigenetic changes in genes regulating lipid metabolism, mitochondrial function and inflammation [57]. DNA methylation in hepatic tissue may mediate these effects, priming metabolic dysfunction before clinically apparent disease [57, 61].

Strong evidence supports a developmental origin of PMOS mediated by in utero androgen excess, often driven by maternal hyperandrogenism, elevated antimüllerian hormone [58, 62, 63] and excessive gestational weight gain [64], which suppress placental aromatase [65, 66] and prolong fetal androgen exposure. This disrupts sexual differentiation and reprogrammes the reproductive and metabolic systems. Daughters of women with PMOS show a higher PMOS risk and metabolic dysfunction [63, 67], while sons exhibit an elevated cardiometabolic risk [68–70]. A landmark mouse study demonstrated that prenatal androgen exposure induces PMOS-like traits through three generations via altered oocyte transcriptomes and sperm non-coding RNAs [63, 67], establishing germline epigenetic inheritance. Notably, some epigenetic and transcriptomic markers are detectable in the serum of first-generation daughters and sons of women with PMOS [63, 67], reinforcing the role of intrauterine and transgenerational programming.

In GDM and type 2 diabetes, intrauterine hyperglycaemia, overnutrition and a sedentary lifestyle act as key environmental drivers, increasing the risk of early-onset MASLD in both daughters and sons [71]. Moreover, epigenetic alterations, such as methylation of *PPARGC1A*, *INS* and *GLUT4* (also known as *SLC2A4*), have been found in muscle, liver and adipose tissue of individuals with type 2 diabetes [60, 72–74] and PMOS [75, 76], linking early exposures to long-term metabolic impairment. These changes are partly reversible through interventions such as exercise [77, 78], highlighting epigenome plasticity. In type 1 diabetes, sons of affected mothers show early vascular dysfunction independent of metabolic changes [79], supporting maternal disease status as a vascular programming signal.

While PMOS shows the strongest inherited epigenetic risk, overlapping mechanisms across MASLD and type 2 and type 1 diabetes point to a shared developmental–epigenomic axis. Future research should map exposure-specific epigenetic signatures for early biomarker discovery and prevention.

## Common pathophysiological mechanisms linking MASLD, PMOS and type 2 diabetes in women

**Fig. 2:**
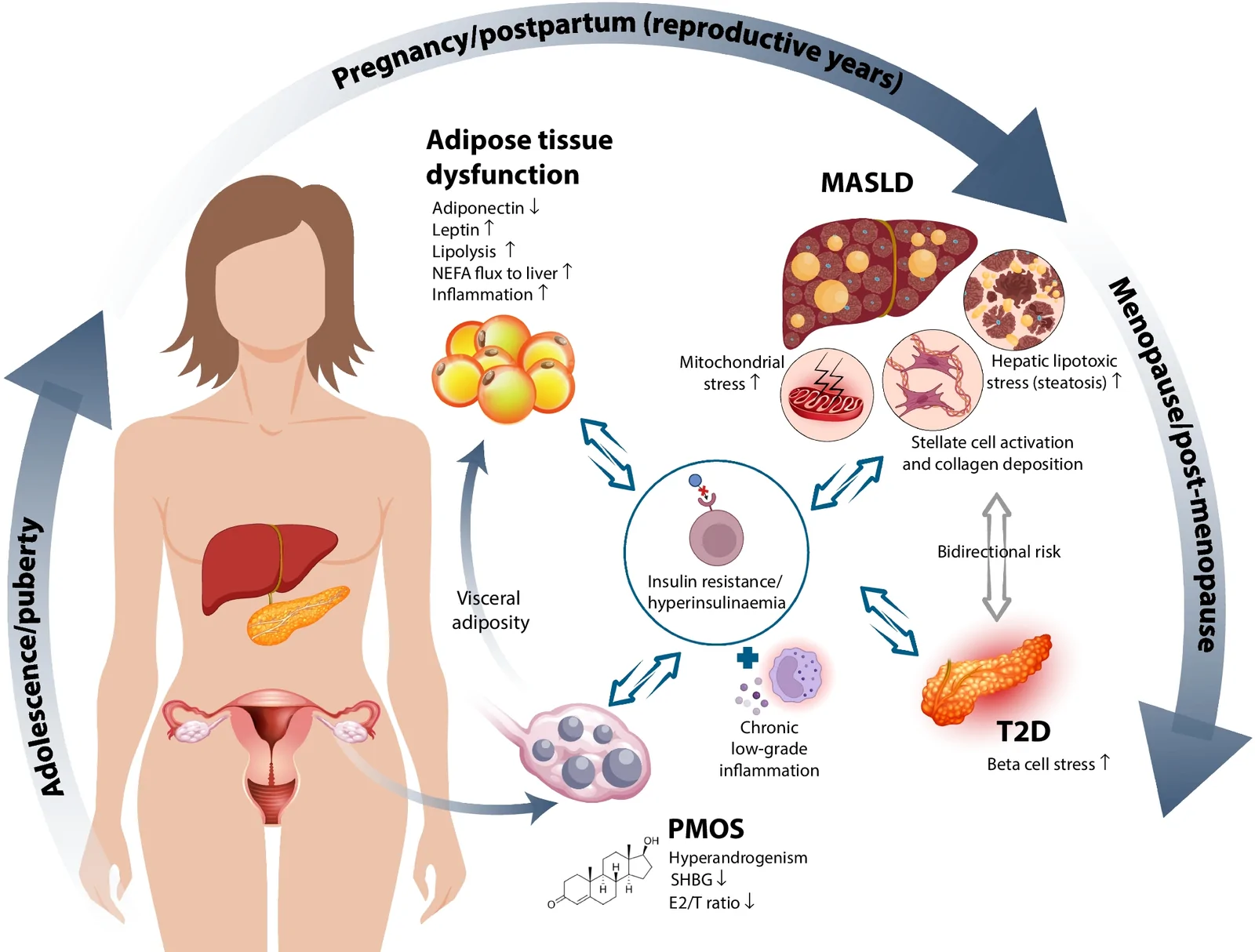
Conceptual schematic illustrating how insulin resistance with compensatory hyperinsulinaemia acts as a central hub linking hyperandrogenism, adipose tissue dysfunction and hepatic steatosis. Hyperinsulinaemia amplifies ovarian androgen production and lowers SHBG, while androgen excess and insulin resistance promote visceral adiposity and adipose dysfunction. Insulin-resistant adipose tissue increases NEFA flux to the liver and alters adipokine/inflammatory signalling, promoting hepatic lipid accumulation and lipotoxic/mitochondrial stress, which can progress to stellate cell activation and collagen deposition. These processes converge on systemic metabolic deterioration, increasing beta cell stress and the risk of type 2 diabetes, while MASLD and type 2 diabetes reinforce each other through shared insulin resistance and inflammatory mechanisms. The outer ring highlights life-stage modifiers—adolescence/puberty, reproductive years (including pregnancy) and menopause/post-menopause—during which shifting sex-steroid milieus and cumulative metabolic exposures modulate pathway strength and disease trajectories. Importantly, hyperandrogenism, adipose tissue dysfunction and type 2 diabetes do not arise exclusively within a single life stage but may emerge at different time points depending on phenotype (e.g. lean vs obese PMOS), genetic predisposition and environmental or metabolic exposures. The schematic therefore represents overlapping and progressive processes rather than strictly stage-restricted events. E2/T ratio, 17β-oestradiol-to-testosterone ratio; T2D, type 2 diabetes. This figure is available as part of a downloadable slideset

The pathophysiological overlap among MASLD, PMOS and type 2 diabetes includes shared drivers such as chronic hyperinsulinaemia, central adiposity, androgen excess, mitochondrial dysfunction and systemic inflammation (Fig. 2) [3]. These factors disrupt hepatic lipid metabolism, promote steatosis and contribute to a broader metabolic syndrome phenotype. From adolescence to menopause, the MASLD–PMOS–type 2 diabetes axis is modulated by shifting hormonal landscapes and cumulative metabolic exposures [80]. Adolescence marks the emergence of insulin resistance and hyperandrogenism. During the reproductive years, increased hepatic lipid burden and insulin demand accelerate metabolic stress. In menopause, decline in oestrogen exacerbates hepatic injury, advancing fibrosis, steatohepatitis and type 2 diabetes [80].

### Sex hormones

In premenopausal women, 17β-oestradiol (E2), primarily ovarian in origin, protects the liver by regulating lipid metabolism, suppressing inflammation and promoting hepatocellular regeneration [2]. In women of all ages, androgens also influence hepatic metabolism, with elevated free testosterone levels doubling the risk of MASLD and metabolic dysfunction-associated steatohepatitis (MASH) [32, 81]. This is linked to decreased sex hormone-binding globulin (SHBG) levels [11, 82], impaired VLDL export of hepatic triacylglycerols and/or fatty acid oxidation, exacerbating fibroinflammation [83]. Recently it was shown that hyperandrogenic women with PMOS are more likely to have hepatic steatosis and greater fibroinflammation [81]. MASLD and PMOS, although complex and multifactorial, share overlapping mechanisms—insulin resistance, hyperandrogenism and adipose tissue dysfunction—all of which increase the risk of type 2 diabetes [84] (see Text box, Androgens and hepatic fibrosis in women: emerging evidence).

**Figure:**
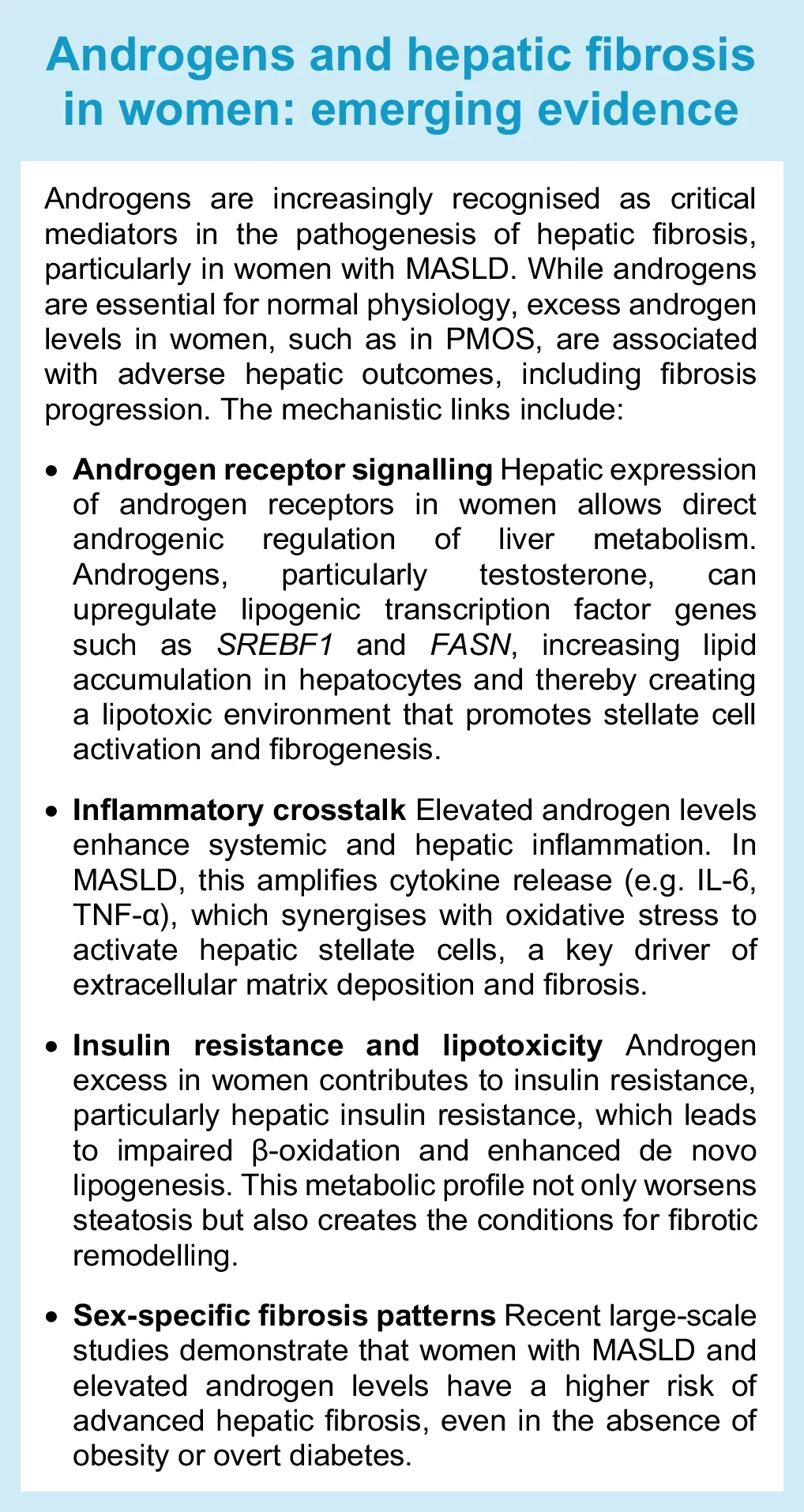

### Insulin resistance

In MASLD, hepatic and skeletal muscle insulin resistance exist even in individuals without obesity and diabetes, impairing gluconeogenesis and de novo lipogenesis and promoting lipolysis [85]. In PMOS, hyperinsulinaemia and insulin resistance are present in up to 90% of women with obesity and 60% of lean women [11, 21], often selectively affecting skeletal muscle, adipose tissue and the liver while sparing the ovaries and adrenal glands [16, 86–88]. This allows insulin to synergise with luteinising hormone in stimulating cytochrome P450 17A1 (CYP17A1)-mediated androgen biosynthesis in ovarian theca cells [12, 89]. Hyperinsulinaemia also suppresses hepatic SHBG production, increasing free testosterone, a key driver of both MASLD and PMOS.

### Adipose tissue dysfunction and inflammation

Emerging evidence supports adipose–liver endocrine crosstalk in PMOS, including altered hepatokines [90] and adipose androgen generation [91, 92], which may amplify hepatic lipid accumulation and systemic insulin resistance. Insulin-resistant adipocytes promote unregulated lipolysis, increasing NEFA flux to the liver, which enhances steatosis [93, 94]. This aligns with human microphysiological evidence that adipocyte inflammation can act as an upstream driver of hepatic insulin resistance [95]. Hyperandrogenism contributes to visceral fat accumulation and ectopic lipid deposition, including in the liver [11]. Enlarged, insulin-resistant adipocytes release less adiponectin [96] and more leptin and proinflammatory cytokines (e.g. TNF-α, IL-6), which are hallmarks of PMOS [12]. In addition, insulin increases aldo-keto reductase type 1 C3 (AKR1C3) activity in PMOS adipose tissue and boosts local androgen production, reinforcing hyperandrogenaemia [91]. Adipose-derived androgens and altered hepatokine signalling perpetuate hepatic steatosis and systemic insulin resistance, creating a proinflammatory, metabolically inflexible environment that impairs thermogenesis and energy balance [12]. This sets the stage for the development of MASLD and type 2 diabetes.

### Fibrosis

Lower E2, hyperandrogenaemia and insulin resistance, often accompanied by adipose tissue dysfunction and inflammation, promote hepatic lipotoxicity, oxidative stress and fibrosis [81]. Hepatic insulin resistance activates hepatic lipogenesis via sterol regulatory element-binding protein 1c (SREBP1c) and carbohydrate response element-binding protein (ChREBP), while impairing β-oxidation and VLDL secretion [84, 97]. Lipid overload triggers mitochondrial dysfunction and hepatic stellate cell activation, advancing fibrosis [97]. Liver macrophages exacerbate this by secreting proinflammatory cytokines (TNF-α, IL-1β, IL-8, CCL2) and TGF-β [98]. In women with PMOS, androgen receptor signalling in the liver may amplify fibrogenic pathways, linking intrinsic hyperandrogenism with advanced MASLD [2].

Studies show that women with ≥F3 fibrosis (on a scale from 0 to 4, where 4 equals cirrhosis), irrespective of PMOS status/phenotype, have elevated androgen levels, independent of age, reinforcing androgen-driven fibrotic progression (see Text box, Androgens and hepatic fibrosis in women: emerging evidence) [13, 99]. Despite these shared mechanisms, MASLD remains underdiagnosed in general and in women with PMOS, particularly at younger ages [100], and sex- and life stage-specific disease pathways remain poorly understood.

### Progression to type 2 diabetes

Type 2 diabetes risk rises in both MASLD and PMOS because of sustained insulin resistance, impaired beta cell compensation and chronic inflammation [81, 97]. The relationship between MASLD and PMOS is bidirectional [53]. Hepatokines such as fetuin-A and fetuin-B and low SHBG levels contribute to peripheral insulin resistance and inflammation. In response to metabolic stress, insulin-sensitising fibroblast growth factor 21 (FGF21) levels rise, reflecting FGF21 resistance. Conversely, excess androgen levels in PMOS impair glucose uptake and mitochondrial metabolism, further promoting hepatic steatosis [101]. Together, PMOS, MASLD and type 2 diabetes form a tightly interconnected metabolic triad.

A deeper understanding of the shared pathophysiological mechanisms linking these conditions may enable more personalised risk stratification and targeted screening in women, particularly during key reproductive life stages. Such insights could also inform the identification of novel therapeutic targets. Future studies should specifically assess whether existing and emerging biomarkers perform equally well in women and men across the life course and whether sex- and age-specific approaches are needed to optimise prediction, prevention and treatment.

## Non-pharmacological and pharmacological interventions

Non-pharmacological strategies remain the first-line interventions for MASLD, PMOS and type 2 diabetes, with substantial evidence supporting dietary changes, physical activity and weight reduction. A sustained 5–10% reduction in body weight significantly reduces liver fat and improves histological features in MASLD [7]. In PMOS, lifestyle modification improves ovulatory function, reduces androgen levels and mitigates metabolic risk [11], while, in type 2 diabetes, lifestyle optimisation delays disease progression and enhances glycaemic management [7]. Nutritional strategies, such as the Mediterranean diet or low glycaemic index plans, show particular benefit in reducing hepatic fat accumulation and improving metabolic markers in MASLD, PMOS and type 2 diabetes [7].

Metformin, although not liver specific, remains the most commonly used insulin sensitiser in type 2 diabetes and is used off label in PMOS. It activates hepatic AMP-activated protein kinase (AMPK), resulting in reduced hepatic gluconeogenesis, improved insulin sensitivity and modest weight loss. However, metformin does not directly reduce hepatic fat or fibrosis in MASLD [7]. Only two drugs are approved for MASLD, specifically for individuals with a clinical profile consistent with steatohepatitis and fibrosis stage 2–3: resmetirom, a liver-directed, thyroid hormone receptor β-selective agonist [102], and subcutaneous semaglutide, a glucagon-like peptide-1 receptor agonist [103]. These drugs are used alongside general lifestyle interventions. Sex-specific analyses in the Phase III trials of resmetirom and semaglutide [102, 103] showed similar treatment responses in men and women. For PMOS, in addition to pharmacotherapy aimed at improving insulin sensitivity, treatment should focus on lowering androgen levels with, for example, combined oral contraceptives [104]. Bariatric surgery improves steatosis in 70–80% of individuals, reducing inflammation and fibrosis [7]. It remains unclear whether targeted treatment of MASLD or PMOS results in improvement of the other condition.

## Screening for liver disease in diabetes and PMOS across the lifespan

Metabolic dysfunction often precedes overt liver disease and recent joint guidelines from EASL, EASD and EASO recommend risk-based assessment for MASLD and advanced fibrosis in individuals with metabolic risk factors, including type 2 diabetes [105]. However, uptake of these recommendations in clinical practice has been limited, partly due to concerns about the performance of non-invasive tests and the risk of over-referral. Importantly, evidence is lacking on the effectiveness of guideline-based assessment strategies in younger women and in female-specific conditions such as PMOS [21] and further research is needed to determine whether such approaches reduce long-term liver-related outcomes.

## Epidemiology of MASLD during pregnancy

In parallel with the rising prevalence of MASLD in the general population, the proportion of pregnancies complicated by MASLD has increased substantially, nearly tripling in the USA between 2007 and 2015 [23]. This trend is concerning, as MASLD during pregnancy is associated with markedly increased risks of adverse maternal and perinatal outcomes, including a threefold higher risk of GDM and higher risks of pre-eclampsia, Caesarean delivery, preterm birth and low birthweight [3]. Emerging evidence further suggests that maternal MASLD may increase the risk of MASLD in offspring [3].

GDM itself is associated with adverse pregnancy outcomes and carries important long-term health consequences for both mother and child, including increased risks of obesity, premature CVD and MASLD. Notably, women with PMOS have a twofold increased risk of developing GDM [106]. Approximately one-third of women with GDM develop type 2 diabetes within 15 years, with the highest risk among women from Central/South America, Africa and the Middle East [107]. In PMOS, Asian and South Asian women develop GDM at lower BMI thresholds, supporting international guidance recommending ethnic-specific BMI and gestational weight gain cut-offs [108].

Established risk factors for progression to type 2 diabetes include higher postpartum BMI and a family history of diabetes [109]. It has also been shown that MASLD amplifies the already elevated risk of type 2 diabetes in women with prior GDM, acting as an additional and potentially modifiable metabolic risk factor [18].

Together, these findings indicate that pregnancy and the postpartum period are critical windows for long-term metabolic risk stratification. In women with prior GDM, with and without PMOS, MASLD assessment may enhance prediction of future type 2 diabetes beyond traditional risk factors such as BMI and family history. Preventive strategies should emphasise lifestyle-based weight management, with incretin-based pharmacotherapy considered for high-risk individuals.

## Convergent pathophysiology of MASLD, PMOS and type 2 diabetes in pregnancy

### Maternal outcomes

Pregnancy reveals and amplifies the shared metabolic and endocrine disturbances of MASLD, type 2 diabetes and PMOS, creating a maladaptive maternal–placental environment that increases the risk of GDM and pre-eclampsia [110, 111]. The placenta regulates nutrient transfer, endocrine output and inflammatory factors in response to maternal metabolic status [112, 113]. In obesity, MASLD, PMOS and insulin-resistant states, elevated maternal insulin activates placental mTOR and increases glucose and amino acid transport, promoting fetal overnutrition [65, 113–115]. Proinflammatory cytokines such as IL-6, TNF-α and leptin further drive trophoblast lipid accumulation and upregulate lipid and amino acid transporters [111], while a lipotoxic placental environment impairs angiogenesis and promotes oxidative and inflammatory stress [115].

MASLD and PMOS both feature hepatic steatosis, insulin resistance and chronic inflammation, which amplify the physiological insulin resistance of late gestation driven by placental lactogen, placental growth hormone, progesterone and TNF-α [116, 117]. In MASLD, impaired hepatic insulin signalling increases gluconeogenesis and hepatic glucose output [110, 111, 118], while PMOS contributes to androgen-driven visceral adiposity, accelerated lipolysis and reduced SHBG, all major predictors of GDM and early-onset pre-eclampsia [119, 120]. These defects intensify placental insulin resistance, disrupt trophoblast mitochondrial function and angiogenesis, and impair spiral artery remodelling [121]. Hepatic steatosis further disrupts glucose homeostasis by amplifying inflammatory signalling and beta cell stress [121]. Concurrent systemic inflammation, altered regulatory T cell responses, endothelial dysfunction and increased coagulation via MASLD-related increases in fibrinogen and factor VIII [110], coupled with PMOS-related PAI-1 excess [122], reduce placental perfusion and gas-exchange efficiency. In addition, women with MASLD have a higher likelihood of Caesarean delivery, with increased rates of both elective and emergency procedures reported across cohorts [123, 124], likely reflecting the higher burden of obstetric complications and fetal overgrowth.

### Fetal and neonatal outcomes

Maternal MASLD is also associated with adverse perinatal outcomes. A cohort study reported an increased risk of preterm birth, with evidence suggesting a relationship with disease severity and comorbid conditions [125]. Furthermore, MASLD has been linked to large-for-gestational-age infants and macrosomia, consistent with maternal insulin resistance and altered lipid metabolism contributing to an adverse intrauterine environment [126].

### Disentangling MASLD from obesity

A key limitation across studies is the difficulty of separating the independent effects of MASLD from those of obesity and related metabolic disorders. MASLD frequently coexists with obesity, insulin resistance and type 2 diabetes, all of which are established drivers of adverse pregnancy outcomes. Although many studies adjust for BMI, residual confounding is likely, particularly in large registry-based analyses [23, 127]. Moreover, MASLD may act both as a mediator and as a marker of underlying metabolic dysfunction. While some data suggest that MASLD confers additional risk beyond obesity, particularly for pre-eclampsia and preterm birth, the magnitude of this independent contribution remains uncertain [123, 128].

Together, these convergent mechanisms underscore the role of MASLD as a marker of metabolic dysregulation affecting both maternal and fetal outcomes. Future studies with detailed metabolic phenotyping and stratification by adiposity are needed to clarify causality.

## Epidemiology of MASLD in menopause

Both the prevalence and severity of MASLD increase markedly after menopause [3, 29]. During the reproductive years, MASLD is generally less common in women than in men; however, this sex difference narrows after menopause, when MASLD prevalence in women approaches or may exceed that observed in men [3]. The menopausal transition is characterised by declining oestrogen levels, weight gain, redistribution of adipose tissue towards a visceral pattern, dyslipidaemia and low-grade systemic inflammation, all of which promote hepatic steatosis and insulin resistance [129]. Consequently, postmenopausal women are at increased risk of both MASLD and type 2 diabetes and tend to present with more advanced liver disease, including greater fibrosis severity [3]. The convergence of central obesity, dyslipidaemia, type 2 diabetes and MASLD further contributes to the loss of the premenopausal cardiovascular risk advantage in women, resulting in a substantial increase in CVD risk after menopause [130]. Together, these changes identify menopause as a critical window for MASLD and type 2 diabetes risk assessment and prevention. Despite these sex-specific disease trajectories, current clinical guidelines do not incorporate sex or menopausal status into risk stratification or treatment decisions.

## Common pathophysiological mechanisms linking MASLD, PMOS and type 2 diabetes in menopause

The postmenopausal state represents a critical convergence point where shared mechanisms among MASLD, PMOS and type 2 diabetes amplify cardiometabolic risk [131]. Oestrogen deficiency drives this interplay by promoting hepatic de novo lipogenesis [132], impairing fatty acid oxidation [133] and reducing lipid export, leading to hepatic steatosis and insulin resistance [134]. This is further exacerbated by the redistribution of adipose tissue from subcutaneous depots to visceral depots [135], increasing portal NEFA flux to the liver [134].

In PMOS, hyperandrogenaemia promotes adipocyte hypertrophy, impaired insulin-mediated suppression of lipolysis and insulin resistance, features that re-emerge or worsen in the hypo-oestrogenic and hyperandrogenic postmenopausal state [11]. Skeletal muscle insulin resistance, a hallmark of both PMOS and menopause, reduces peripheral glucose disposal and diverts carbohydrate substrates towards hepatic lipogenesis [11, 134].

Oestrogen deficiency also contributes to mitochondrial dysfunction and gut microbial dysbiosis, which are both implicated in MASLD progression [134]. At the cellular level, impaired ERα and GPER1 (membrane-bound ER) signalling reduces anti-inflammatory and antifibrotic defences, promoting Kupffer cell M1 polarisation and hepatic stellate cell activation [134].

Genetic susceptibility, particularly *PNPLA3* polymorphisms interacting with hepatic ERα, further increases MASLD risk in postmenopausal women [134]. This convergence of hormonal, metabolic and genetic factors underscores the urgent need for tailored prevention strategies in women with PMOS or type 2 diabetes after menopause.

## Hormone replacement therapy in MASLD prevention

In addition to lifestyle modification, hormone replacement therapy (HRT) is a potential strategy to counteract oestrogen deficiency-driven mechanisms underlying MASLD progression. Several studies report that HRT improves liver enzymes, insulin sensitivity and fat distribution, with transdermal routes showing more favourable effects than oral formulations because of reduced hepatic first-pass exposure [134]. Transdermal oestrogen enhances fat oxidation and may support metabolic flexibility, while certain progestogens appear to worsen lipid metabolism and fibrosis [134]. However, the evidence is heterogeneous and confounded by inconsistent study designs and biases, highlighting the need for rigorous, stratified clinical trials to evaluate HRT’s role in MASLD prevention.

## Human-relevant in vitro models for investigating the MASLD–PMOS–type 2 diabetes axis

**Fig. 3:**
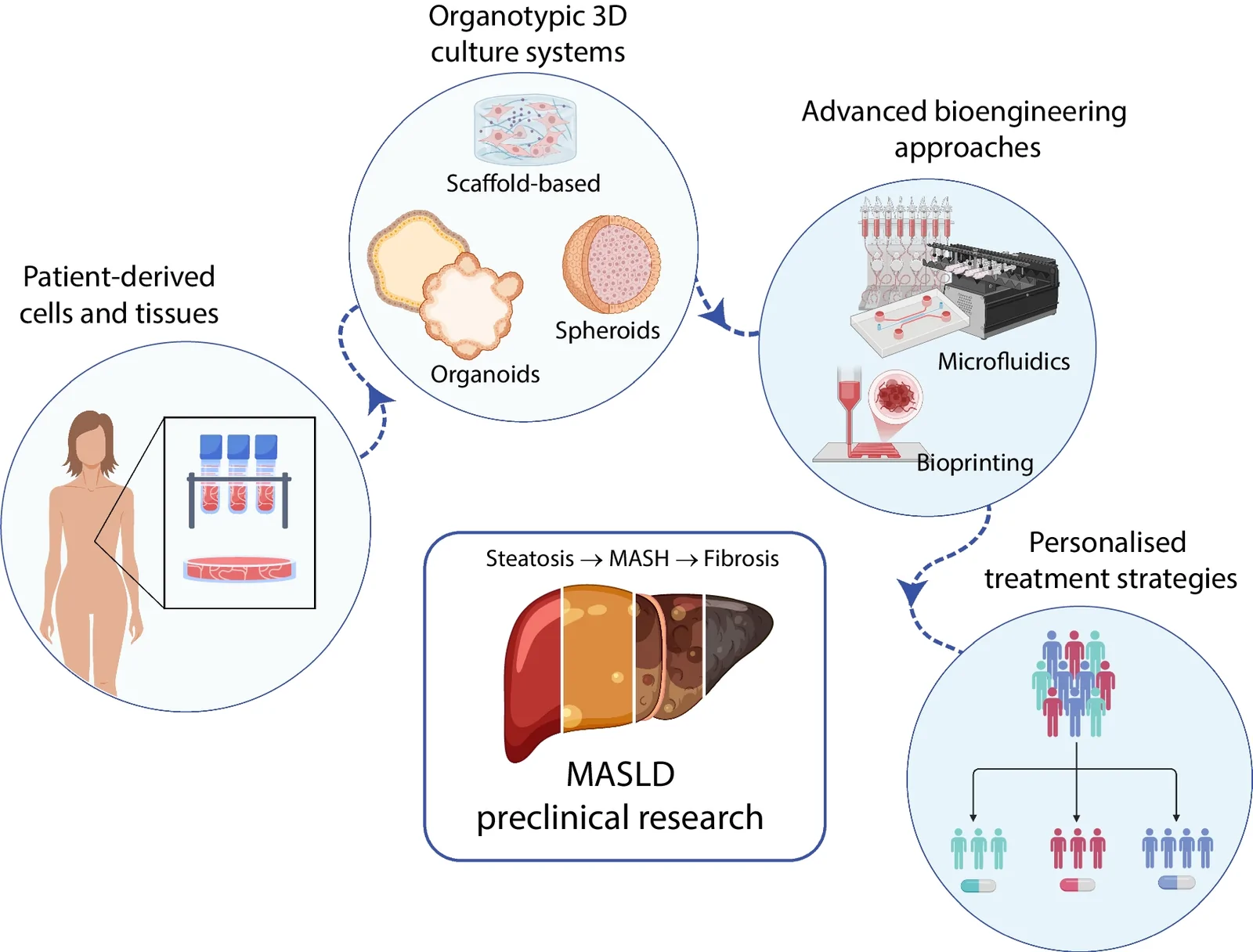
Schematic overview of how patient-derived cells and clinical samples can be integrated with organotypic 3D culture systems (scaffold-based cultures, spheroids, and organoids) and advanced engineering approaches (microfluidics and bioprinting) to generate human-relevant models spanning the different stages of MASLD progression. These platforms enable controlled metabolic and hormonal perturbations, incorporation of multicellular tissue architecture, and dynamic perfusion or tissue–tissue communication, thereby improving mechanistic interrogation and translational predictivity compared with conventional 2D cultures. When combined with longitudinal sampling and multi-parameter phenotyping, such models support the identification of biomarkers and predictors of drug response and facilitate stratification of patient subgroups for targeted therapeutic testing. This figure is available as part of a downloadable slideset

The intersecting pathophysiology of MASLD, PMOS and type 2 diabetes calls for human-relevant models that can capture complex endocrine–metabolic interactions and their modulation by sex hormones across life stages (Fig. 3). In vitro platforms have advanced rapidly, providing the experimental control and flexibility needed to dissect mechanistic pathways under defined hormonal and metabolic stimuli, an essential capability for studying sex-specific risk trajectories that are often incompletely represented in animal models. This has been further accelerated by the growing adoption of new approach methodologies in drug development and by regulatory momentum following the FDA Modernization Act 2.0, which removed the blanket statutory requirement for animal testing prior to first-in-human studies and broadened the acceptance of human-relevant non-clinical evidence [136, 137].

Conventional 2D hepatocyte cultures have long served as foundational tools for probing hepatic responses to metabolic stressors in vitro. However, 2D models are limited by rapid dedifferentiation and loss of mature hepatocyte function and, particularly when relying on hepatic cell lines, by molecular and functional states that diverge from in vivo liver phenotypes. These limitations together constrain their usefulness for modelling chronic, progressive diseases [138].

Moving beyond 2D approaches, organotypic 3D platforms (spheroids and organoids), especially those built from primary human cells or pluripotent stem cell-derived lineages, better preserve tissue architecture and multicellular biology over time [138–141] and enable more physiologically relevant modelling of chronic, slowly progressive conditions such as MASLD, PMOS and type 2 diabetes. By co-culturing primary human hepatocytes with key non-parenchymal cells, these systems can reproduce not only steatosis but also other core disease features including insulin resistance, inflammatory signalling and fibrogenic remodelling, supporting both mechanistic discovery and translational drug testing in a human, disease-intrinsic context [138, 142]. At the reductionist–personalised interface, induced pluripotent stem cell (iPSC)-derived hepatocyte-like cells enable scalable, genotype-aware modelling to interrogate genetic risk and inter-individual variability (e.g. *PNPLA3*-related susceptibility) and to test how metabolic overload and sex hormones jointly shape hepatocyte lipid handling, stress responses and insulin signalling across the female lifespan [141, 143]. However, standardised approaches that robustly recapitulate specific endocrine environments remain under development. In this regard, physiological relevance can be increased by applying controlled hormone exposure paradigms and, where appropriate, by conditioning cultures with well-characterised donor sera (e.g. PMOS vs control) while accounting for metabolic and inflammatory confounders [144].

Another promising and complementary strategy is to move from static ‘single-organ’ models towards dynamic, multi-tissue microphysiological systems (MPS). In recent years, advanced microfluidic and organ-on-chip platforms have been developed to model complex multi-organ interactions in health and disease, enabling stable long-term perfusion cultures, physiological gradients, time-varying exposure schedules and defined tissue–tissue communication that are difficult or impossible to achieve in static cultures [95, 145, 146]. Multi-organ reproductive tract MPS coupled to liver have already demonstrated cycle-like endocrine control over peripheral tissues, providing a conceptual template for introducing PMOS-associated or other endocrine disturbances under controlled conditions [144]. In parallel, an iPSC-derived adipocyte–hepatocyte–macrophage MPS demonstrated that adipocyte inflammation can act as a primary upstream driver of hepatic insulin resistance and steatotic change, highlighting inflammatory adipose–liver coupling as a mechanistically actionable node in metabolic liver disease [95] and a potential highly relevant axis for PMOS-associated metabolic risk.

Together, these advances point towards next-generation organ-on-chip platforms that can ideally integrate reproductive endocrine cues with tissue–tissue crosstalk, enable controlled hormone cycling and support longitudinal sampling, capabilities that are well suited to modelling puberty, pregnancy and menopause within a unified female-lifespan framework.

## Future perspectives and knowledge gaps

Key knowledge gaps and future directions are summarised in the text box. MASLD is an emerging modifier of diabetes risk and prognosis in women, shaped by reproductive transitions such as early menarche, pregnancy, menopause and androgen-excess states such as PMOS. PMOS, characterised by hyperandrogenism and insulin resistance, disproportionately increases hepatic steatosis, fibroinflammation and cardiometabolic risk. Current risk stratification overlooks life-stage metabolic phenotypes, limiting early identification of women at risk for type 2 diabetes and progressive liver disease.

Opportunities for targeted intervention remain underused. Screening should align with reproductive milestones: adolescence in PMOS, peripartum periods after GDM, and the menopausal transition, when declining E2 accelerates hepatic risk. Pregnancy is a critical window when latent vulnerability is revealed, and incorporating MASLD assessment after GDM may improve prediction of future type 2 diabetes. This highlights the need for pragmatic, risk-based screening pathways and sex-specific biomarkers of steatosis and fibrosis.

**Figure:**
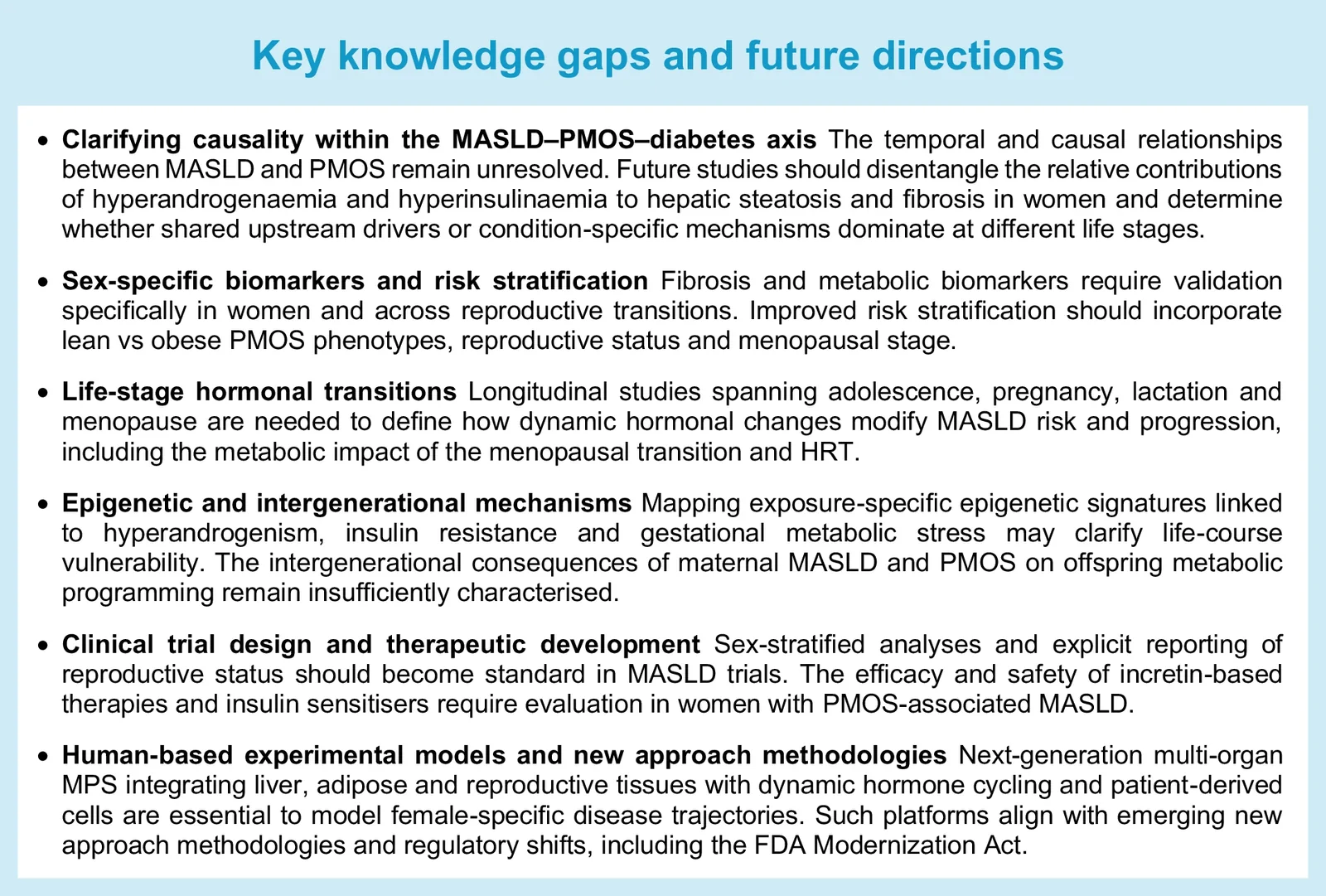

Mechanistic gaps persist, particularly in understanding how fluctuating E2, androgens and pregnancy hormones modulate adipose–liver signalling, insulin resistance and fibrogenesis across reproductive stages. Addressing these gaps requires longitudinal life-course studies and experimental models that integrate sex and reproductive status. Such insights will be essential for designing effective sex- and life stage-tailored prevention strategies for MASLD and diabetes in women.

## Conclusion

The complex interaction between hepatic metabolic regulation and female reproductive function underscores the critical role of the hepato-ovarian axis in women’s health. This bidirectional relationship requires consideration of reproductive stage and hormonal environment—including menarche, pregnancy, menopause and conditions such as PMOS—as central factors in both clinical and experimental research. Including sex and reproductive status as biological variables in preclinical models is essential to uncover the mechanisms underlying sex-specific susceptibility to MASLD. A deeper understanding of these dynamics across the reproductive lifespan will be crucial for advancing precision medicine strategies tailored to the unique metabolic and hormonal profiles of women.

## Supplementary Information

Below is the link to the electronic supplementary material.

Slideset of figures (PPTX 749 KB)

## Abbreviations

E2: 17β-Oestradiol
ER: Oestrogen receptor
GDM: Gestational diabetes mellitus
iPSC: Induced pluripotent stem cells
MASLD: Metabolic dysfunction-associated steatotic liver disease
MPS: Microphysiological systems
PCOS: Polycystic ovary syndrome
PMOS: Polyendocrine metabolic ovarian syndrome
SHBG: Sex hormone-binding globulin
